# Cook Co Hospital

**Published:** 1870-04

**Authors:** 


### *Cook County Hospital.*

At the meeting of the Board of Supervisors of this county, a few days since, the authorities definitely stated that there was room for no more patients in the brick monstrosity below Eighteenth street, and propose to board out patients at "Providence Hospital," wherever that may be.

Is it not about time that some sense should find its way into the noddles of officials?

Let the old hospital be sold for a brewery, or packing-house, and the money be applied to the purchase of *ample* grounds, well drained, and with a chance for the winds to purify the air above, and then put up cheap buildings, and plenty of them.

We repeat: Let us have no more Bastiles of Disease.

